# Prism coupling and visualization of surface nanoscale axial photonic structures

**DOI:** 10.1038/s44172-026-00672-x

**Published:** 2026-06-01

**Authors:** Anatoliy Savchenkov, Zijie Wang, Manuel Crespo-Ballesteros, Michael Sumetsky, Andrey Matsko

**Affiliations:** 1 Jet Propulsion Laboratory, California Institute of Technology, Pasadena, CA USA; 2 Aston Institute of Photonic Technologies, Aston University, Birmingham, UK

**Keywords:** Integrated optics, Nanophotonics and plasmonics

## Abstract

Surface nanoscale axial photonics (SNAP) has demonstrated the record subangstrom precise fabrication of miniature optical devices enabling applications in classical and quantum signal processing unfeasible to date. However, SNAP devices formed by subnanometre protrusions of the optical fiber surface lack robustness and simple identification methods. This complicates their controlled alignment and raises concerns about their practical applicability. Here we introduce techniques that enable both direct visualization of nanoscale SNAP features and efficient coupling to fused-silica SNAP microresonators using evanescent-field prism couplers. These advances provide precise control of light-resonator interactions and establish a scalable and robust route towards on-chip integration of SNAP devices, opening opportunities for their critical applications in computing, telecommunications, ultraprecise sensing, and fundamental optical science.

## Abstract (web summary)

Surface nanoscale axial photonics (SNAP) enables fabrication of miniature optical devices. Anatoliy Savchenkov and colleagues introduce methods for visualization of SNAP features and evanescent prism coupling to the fused-silica nanostructures.

## Introduction

Surface nanoscale axial photonics (SNAP) technology exploits ultrafine nanoscale perturbations in the radius of an optical fiber controlled with subangstrom precision not attainable in any other photonic platform^1^. By carefully engineering these spatial perturbations, it is possible to form circuits of optical cavities with tailored resonant properties. However, SNAP devices demonstrated to date lack robustness and simple identification methods. Here, we introduce techniques that enable efficient coupling to fused-silica SNAP microresonators using evanescent-field prism couplers and direct visualization of nanoscale SNAP features. Taken together, these advances establish an experimentally validated pathway for transitioning SNAP from a laboratory-only platform to a practical microscale photonic technology. The ability to fabricate, visualize, and reliably interface with surface-defined axial photonic circuits at the subangstrom scale is foundational for future applications. These include miniature resonant filters, dispersion-engineered delay lines, compact microwave-photonic components, and potentially elements for telecommunication, optical-computing, and quantum-sensing architectures. These capabilities were previously inaccessible due to the absence of a stable coupling mechanism and the lack of direct structural characterization.

Evanescent field coupling is needed to send light to and retrieve light from a SNAP cavity. The coupling relies on the overlap of the evanescent field from the input waveguide or coupler with the evanescent field of the SNAP modes. To date, SNAP devices have been demonstrated on the basis of the tapered fiber coupling. The tapered waveguides used to provide high efficiency coupling and to allow tunability by mechanical motion of the fiber taper touching the surface of the SNAP waveguide.

Among the various methods developed for exciting dielectric resonators, tapered optical fibers are known to be the most effective^2–10^. In this approach, a standard single-mode fiber is adiabatically thinned down to a waist diameter of only a few micrometers, which is well matched to near-infrared light. When positioned in close proximity to the resonator’s surface, the tapered region enables efficient evanescent coupling. This geometry simplifies both the injection of light into the resonator and the extraction of the transmitted or scattered signal. By adjusting the taper position and polarization, one can preferentially excite specific resonator mode families. Experiments have demonstrated that, for high-quality fused silica resonators, the external coupling efficiency achievable with this method can approach unity, with values reported as high as 99.99%^7^.

The standard optical fiber taper configuration has zero airgap between the coupler and the SNAP cavity (the taper is attracted to the cavity because of residual surface charging), and is useful due to its simplicity of implementation that does not require visual alignment of the cavity and the coupler. The coupler can be pulled along the optical fiber with SNAP cavities built on it until the cavity becomes identifiable via the change of the optical transmission of the system. On the other hand, the tapered fibers are delicate and sensitive to vibrations, dust, or temperature fluctuations.

Limitations of tapered fibers can be eliminated by prism coupling that uses total internal reflection within a high-index prism to generate an evanescent field at the interface. The coupling technique was initially developed for optical waveguides^11–15^ and then was applied to microresonators^16–21^. Light can efficiently tunnel into the cavity from the prism coupler when the incident angle of the input optical beam satisfies phase-matching conditions. A wide range of wavelengths can be efficiently coupled by adjusting the angle. Prism coupling is challenging to realize with SNAP structures because the coupling requires precise alignment and calls for visualization of the cavity. It also involves a free optical beam that has to be optimally shaped. On the other hand, the coupling technique can provide high mechanical stability, power handling, and thermal robustness of the structure and is promising for robust packaging of SNAP structures.

SNAP structure has less than a nanometer radial contrast and tens of microns length. A special technique has to be developed to visualize the resonators to adjust a prism coupler. We optimized the dark field imaging technique for this purpose. It was found more than a century ago that the separation of the illumination and detection pathways enabled the observation of nanoparticles^22^. The technique, dubbed dark field microscopy, was advanced since then, allowing detection of low constant particles approaching 20 nm in size (see ref. ^23^ for review). Nanofibers have also been utilized as an effective tool to detect single nanoparticles due to the scattering induced by them^24^.

We here adapted the direct visualization methods developed for the nanoscale particles^25^ for the identification of the geometrical position of the structural variations of the cylindrical fibers. These variations are responsible for the SNAP formation. Because of the resonant properties of SNAP resonators, we were able to clearly detect their position even though they had extremely small contrast. Visualizing the cavities and coupling an external coherent pump to them using an evanescent field prism coupler, we demonstrated undercoupled, critically coupled, and overcoupled operational regimes of the system. We also compared SNAP spectroscopy techniques using tapered fiber being in full contact with the SNAP structure and prism couplers separated from the SNAP by a finite air gap and demonstrated that the prism coupler can provide better spectral resolution of the measurements if compared with the particular realization of the fiber taper we utilized. The demonstrated technique is promising for future applications of the SNAP cavities in robust and compact photonic assemblies.

## Methods

### Experimental setup

**Fig. 1:**
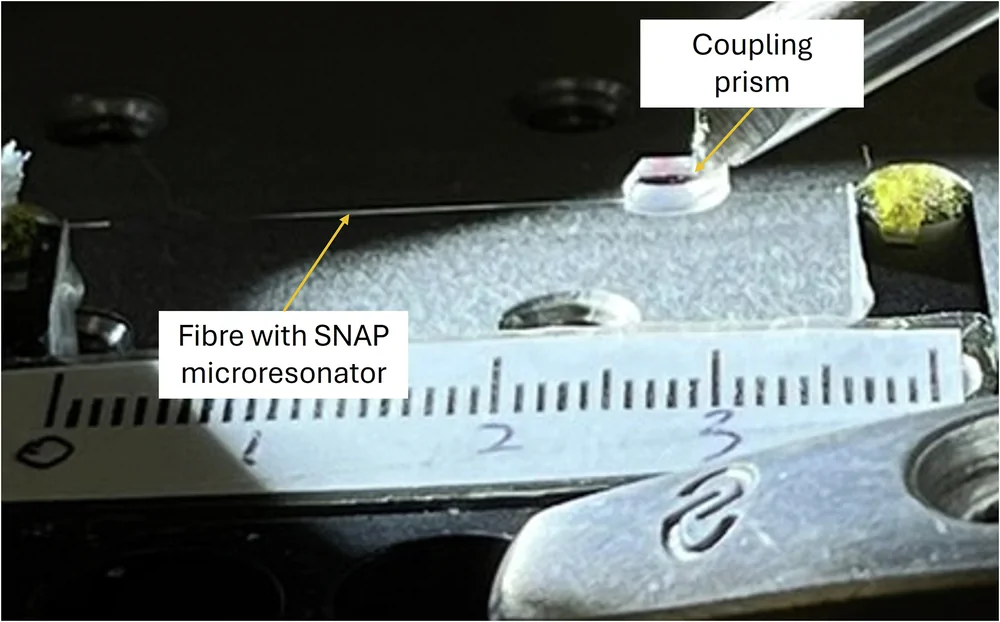
Experimental setup photo. SNAP cavity coupled using a prism coupler.

The prism coupler Fig. 1 allows achieving tunable coupling with the SNAP cavity. The coupling realization is hard because it requires visualization of the SNAP cavity for the good alignment.

**Fig. 2:**
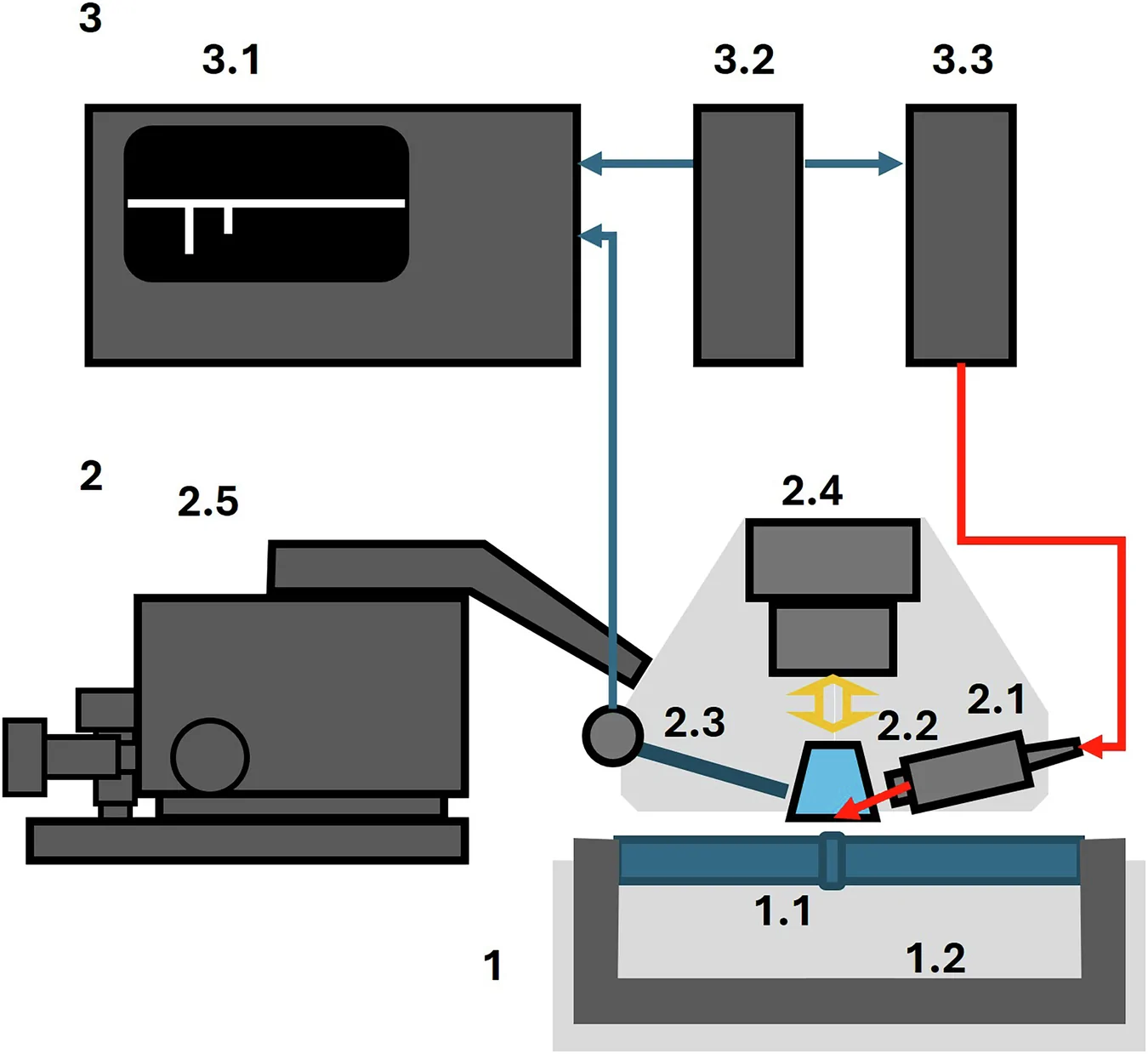
Experimental setup schematics. Experimental setup with prism coupler includes three major subassemblies with components denoted as “1.x” for the cavity subassembly (“1.1” is the SNAP cavity), “2.x” for the controlled prism coupler subassembly (“2.5” is the translation stage), and “3.x” for the optical scalar network analyzer subassembly (“3.3” is the laser). The detailed description of the subassemblies is presented in the text.

The experimental setup based on the prism coupler to the SNAP cavity (Fig. 2) is created as follows. The frequency of a narrowband laser 3.3 is saw-tooth-modulated with the function generator 3.2. The input of an oscilloscope 3.1 receives the output of an optical photodiode 2.3. The oscilloscope sweep is synchronized with the function generator. Therefore, the oscilloscope shows full optical transmission between the output of the laser and the photodiode.

Optical SNAP cavity 1.1 is mounted on a metal U-bench 1.2 and evanescently coupled to BK7 custom-shaped prism 2.2. The prism 2.2, the collimator 2.1, the output waveguide with the 2.3, and the interferometric optics measuring the prism-resonator airgap 2.4 form a single prealigned subassembly, whose position is controlled by a motorized translation stage 2.5. The motorized stage 2.5 controls the air gap of the evanescent coupling 2.2–1.1 and the position of the coupling along the fiber. To identify eigenfrequencies of the mechanical oscillations of the suspension of the SNAP cavity 1.1.

## Results

### Optical visualization of SNAP cavities

**Fig. 3:**
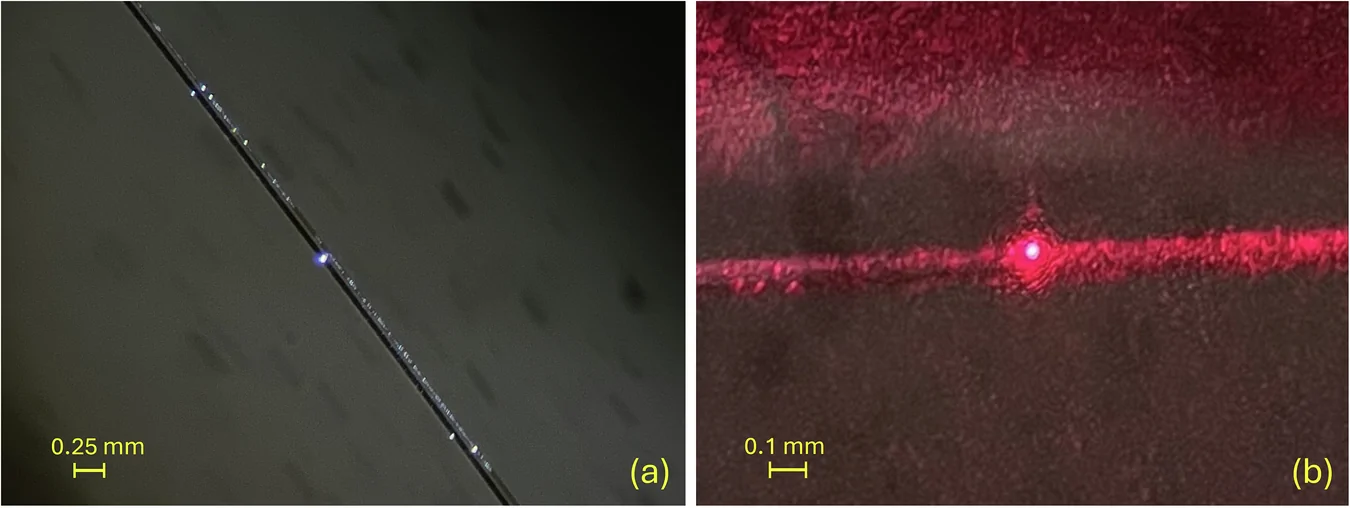
Optical images of the SNAP resonators. **a** High-contrast dark-field imaging of low-contrast ring cavities on an optical fiber surface. A bright white LED coupled to the fiber tip excites multiple waveguide modes, including higher-order modes that are well suited for interacting with the SNAP resonator. The LED's broad optical spectrum ensures the excitation of a wide range of waveguide modes, which in turn can efficiently couple to the leaky modes of the resonator. Background light is suppressed via spatial filtering between the LED source and fiber. This technique enhances contrast, enabling clear visualization of the subtle ring cavity structures shown. **b** Identifying a low-visibility SNAP cavity location via guided light termination. Coherent red light is evanescently coupled into the fiber using a mode-matched coupler. In this case, the lower order waveguide modes are not excited. The cavity serves as a distinct boundary, both reflecting and attenuating the guided light due to presence of high order leaky modes. Coupling light sequentially to either side reveals the cavity’s position as the sharp cutoff point where the leaky mode is localized. The scale of the picture is given by the 125 μm diameter of the fiber. The radial contrast of the SNAP resonator does not exceed 0.2 nm.

To realize the prism coupling, we developed techniques for visualization of the nanoscale structures. The technique is either based on standard dark-field microscopy or optical resonant scattering of confined laser field. In the case of dark field microscopy the SNAP waveguide was illuminated with a collimated white light source and the scattered light was recorded using a microscope collecting the light scattered orthogonally to the direction of the collimated light (Fig. 3a). In the case of the coherent field scattering the light from a red laser was coupled to the SNAP waveguide and the scattering by the SNAP structure was observed by a microscope equipped with a camera (Fig. 3b). The optical beams coupled to the fiber using a prism coupler and propagating in the SNAP preform with high orbital momentum were coupled to the leaky SNAP modes. The scattering of the light confined in the modes was detected. These two techniques allowed clear observation of the nanoscale SNAP objects.

The later idea of visualization of the nano-objects is based on the availability of the leaky SNAP modes that are coupled with the whispering gallery modes of the fiber. The coupling is related to the generation of the high angular momentum beams using fused silica microspheres mounted on waveguides^26–28^. In the case of visualization one excites the high-order waveguide modes using a frequency chirped laser that results in excitation of the SNAP cavities when the frequency of the laser hits the frequency of the mode. The visualization appears because the excitation beam is confined in the waveguide and does not contaminate the field of view. The leaky SNAP modes also emit to free space as a result of light confining geometry of SNAP as well as micro-defects naturally present in the system. The emission is bright because of the high concentration of light in the cavity.

### Prism coupling to SNAP cavities

The operating mechanism of a prism coupler relies on frustrated total internal reflection. When light is totally internally reflected at the base of a high–index prism, an evanescent field is established at the interface. If a dielectric ring resonator is brought into the near field of this interface, the evanescent tail overlaps with the resonator field, allowing energy transfer. For efficient coupling, the in-plane wave vector component of the prism mode must match the azimuthal propagation constant of the ring resonator. For example, this condition, known as phase matching for a whispering gallery mode resonator, is expressed as

1 $${k}_{0}\sin \theta \approx \frac{m}{{n}_{prism}{R}_{in-plane}},$$

where *m* is the azimuthal mode number, *R*_*in*−*plane*_ the resonator radius in plane with the coupler, and *θ* the incidence angle inside the prism^19^. The same condition should be satisfied to couple to a SNAP cavity.

The choice of prism material is determined by the need to provide a sufficiently large refractive index to reach the mode effective index. For silica microspheres and toroids, glass prisms such as BK7 or SF11 are commonly used, while high-index resonators based on lithium niobate, magnesium fluoride, or semiconductors often require rutile (TiO_2_) or gadolinium gallium garnet (GGG) prisms^29,30^. In birefringent prisms, the orientation can be adjusted to achieve polarization-selective coupling, enabling discrimination between TE and TM modes^30^. We used glass as the prism host material.

Efficient prism coupling requires not only phase matching but also a high spatial overlap between the incident optical field and the resonator mode. The fundamental modes of a ring dielectric cavity have a quasi-Gaussian field profile in the radial direction and are confined near the equatorial plane of the resonator. The circulating field extends azimuthally with a lateral width proportional to $\sqrt{Rh}$, where *R* is the resonator radius of curvature and *h* the scale of the evanescent field proportional to the operating wavelength^19^. To maximize coupling, the incident beam should be shaped to match this effective mode size and divergence.

A Gaussian beam is typically used for coupling purposes. The beam is focused to a waist that approximately matches the mode waist at the resonator–prism interface. Over-focusing results in a beam with high divergence, which reduces the overlap integral due to mismatch in angular momentum. Conversely, an under-focused beam interacts with a larger resonator surface region, decreasing spatial overlap and introducing coupling to higher-order modes. Optimal efficiency is achieved when the waist is positioned close to the prism interface and aligned with the equatorial plane of the resonator.

**Fig. 4:**
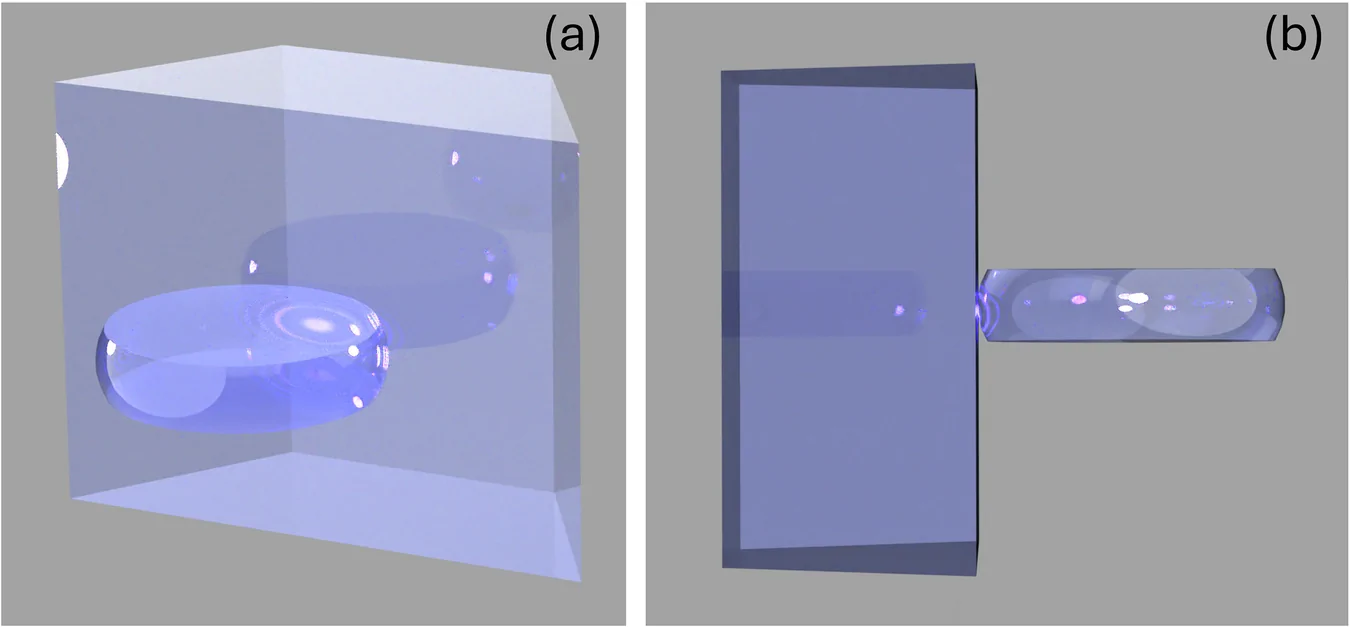
Prism coupling to a whispering gallery mode resonator. **a** 3D view of the coupling. The coupling aperture defined by the resonator shape looks as an elliptic spot. **b** Side view of the prism and the resonator.

Nearly ideal aperture matching of a dielectric resonator with a circular Gaussian beam occurs when the resonator is shaped properly^31,32^. For example, for a spheroidal resonator (Fig. 4), the vertical radius of the spheroid has to be optimized so that

2 $$\cos \theta =\sqrt{\frac{{R}_{orthogonal}}{{R}_{in-plane}}}$$

where *R*_*orthogonal*_ is the vertical radius of curvature of the resonator in the vicinity of the contact spot. Instead of adjusting the resonator shape one should be able to adjust the shape of the beam to match one leaving the resonator.

The performance of a prism coupler is inherently constrained when planar prism surfaces are employed, as they neglect the curvature of the resonator surface. A proposed solution is the use of concave prism interfaces with radii of curvature designed to approximate that of the resonator^19^. Nevertheless, the fabrication of accurately curved prisms and the realization of efficient optical coupling remain technologically challenging.

The coupling strength is governed primarily by the resonator–prism gap. Since the evanescent field decays exponentially with distance, small changes in the gap lead to large variations in the external coupling rate. This is usually quantified through the coupling quality factor *Q*_c_, which combines with the intrinsic cavity losses to define the loaded linewidth. Critical coupling, in which all incident power is transferred to the resonator, occurs when *Q*_c_ equals the intrinsic quality factor *Q*_0_. The strong exponential dependence of the coupling rate on the gap makes nanopositioning stages with sub-100 nm resolution essential^31^.

The loading quality factor in the first approximation follows the exponential law^31^

3 $${Q}_{c}={Q}_{c0}{e}^{d/h},$$

where *d* is the air gap between the surfaces of the resonator and the surface of the prism, *h* is the evanescent field parameter; *Q*_*c*0_ is the quality factor at zero spatial gap, *d* = 0. The value of zero-gap loaded quality factor depends on the geometry of the system and refractive indexes of the prism and the resonator. The evanescent field parameter can be defined as

4 $$h=\frac{\lambda }{4\pi \sqrt{{n}_{SNAP}^{2}-1}},$$

where *λ* is the optical wavelength, *n*_*SNAP*_ is the refractive index of the resonator. This parameter does not depend on the morphology of the resonator.

The relative amplitude of the field of the light exiting the resonator in the case of the perfect mode matching is

5 $$R(\omega )=\frac{{Q}_{c}-{Q}_{0}-2i{Q}_{c}{Q}_{0}(1-{\omega }_{0}/\omega )}{{Q}_{c}+{Q}_{0}-2i{Q}_{c}{Q}_{0}(1-{\omega }_{0}/\omega )},$$

where *ω*_0_ and *ω* are the frequencies of the SNAP mode and the light. The resonant power reflection coefficient ∣*R*(*ω* = *ω*_0_)∣^2^ is zero when *Q*_*c*_ = *Q*_0_. This is the case of critical coupling. In more realistic case of incomplete mode matching, the reflection amplitude coefficient for the output light is $\widetilde{R}={\xi }_{1}+{\xi }_{2}R$, where *ξ*_1_ and *ξ*_2_ are normalized frequency dependent complex numbers (∣*ξ*_1_∣^2^ + ∣*ξ*_2_∣^2^ = 1).

**Fig. 5:**
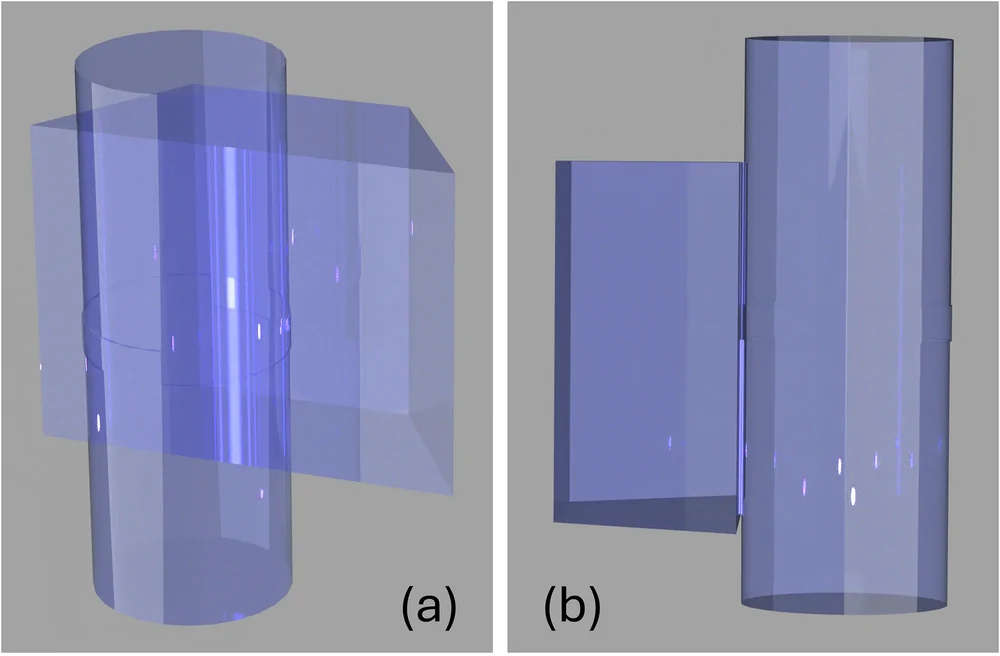
Standard prism coupling to a SNAP resonator. **a** 3D view of the coupling. The coupling aperture defined by the resonator shape looks as a set of lines. **b** Side view of the prism and the optical fiber. The SNAP structure has negligibly small contrast.

Coupling to a SNAP cavity using a standard prism coupler (Fig. 5) is problematic because the SNAP profile does not exceed a few nanometers. With respect of the prism coupler, SNAP structure is just a cylinder. The coupler–waveguide assembly (Fig. 5) has a rectangular-linear aperture in this case (shown by while line in Fig. 5a). It is possible to couple to a SNAP cavity through this aperture by shaping the coupling beam optimally for the SNAP mode profile, however the beam also will couple with the continuum of modes of the cylindrical preform. The phase matching is not selective. The selective coupling to the SNAP modes is rather involved. Also, it is nearly impossible to align the surfaces of the cylinder and the flat prism.

**Fig. 6:**
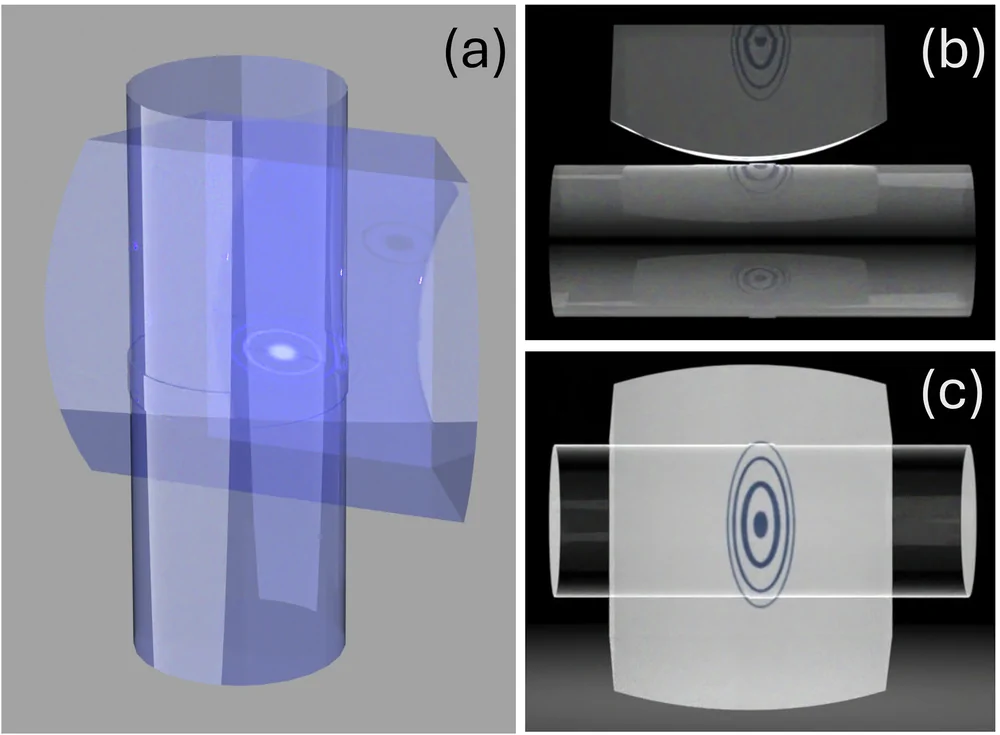
Curved prism coupling to a SNAP resonator. **a** 3D view of the coupling. The coupling aperture defined by the resonator shape looks as an ellipse. **b** Side view of the curved prism and the optical fiber. **c** View of the coupling aperture through the prism. In experiment the aperture can be characterized by the Newton rings.

We realized efficient prism coupling with the SNAP cavity, taking advantage of our ability to visualize it. For better phase matching selectivity the prism was produced out of a cylindrical lens (Fig. 6). The cylindrical shape of the prism facet improved the phase matching with the nano-bump on the waveguide and simplified the alignment procedure. The curved interface prism has an elliptical coupling aperture, where one axis of the ellipse is given by the curvature of the cylinder, and the other—by curvature of the prism. The main idea is that in the case of the curved prism the prism curvature replaces the curvature of the cavity in the case of optimal coupling to a whispering gallery mode resonator. Equation (6) should be rewritten as

6 $$\cos \theta =\sqrt{\frac{{R}_{prism}}{{R}_{in-plane}}}$$

Since we know approximately the shape of the SNAP mode, we can select the prism curvature radius *R*_*prism*_ to match one of the SNAP modes. The coupling beam also be modified to match the curvatures. All these preparations are doable since the prism can be cut out of a cylinder preform with the optimal curvature.

To find relationship between the curvature of the prism and the beam radius, we introduce the evanescent field scale as parameter *h*, the “*e*^−2^” geometrical width of the mode of a particular SNAP cavity under test as a parameter *w*, and the incident angle of the incoming light as *θ* (defined by Eq. (2)). In this case

7 $${R}_{prism}\approx \frac{{w}^{2}}{{8h}\,{\cos }^{2}\theta }.$$

The optimal curvature radius ensures forming a symmetrical Gaussian beam in the prism’s far-field, which is easy to couple with standard optical collimators.

**Fig. 7:**
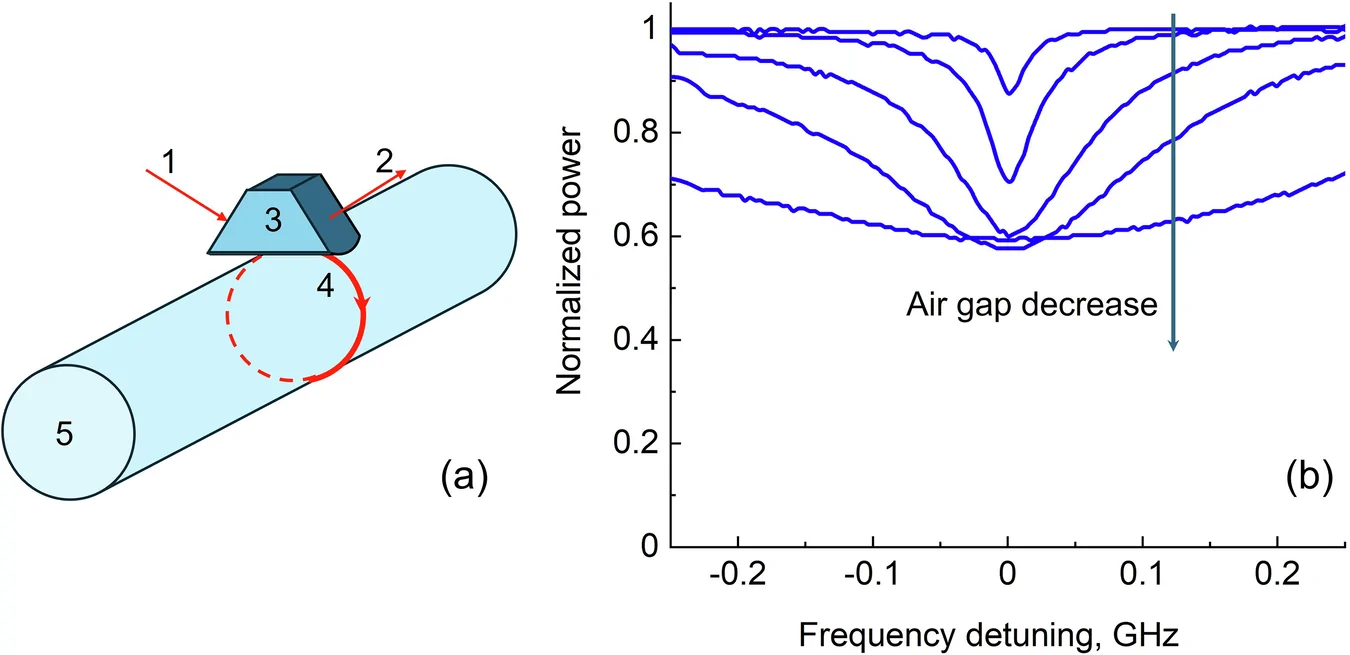
SNAP resonator coupling and its spectra. **a** Schematic of the coupling setup: an input focused laser beam (1) evanescently couples light into a specially designed SNAP structure (4) via BK7 prism (3), curved at its surface facing the hosting fiber (5). The light interacts with the resonator (4) and leaves (2) it through the same prism (3). The transmitted power is collected after the interaction. **b** Normalized transmission spectra of the SNAP resonator as the gap between the focused beam’s waist and the SNAP structure is progressively reduced (indicated by the arrow). To achieve it the beam waist is focused on the curved prism surface, and the SNAP structure is moved closer to it so that no refocusing of the beam is needed. The curve exhibiting the lowest extinction at resonance corresponds to the undercoupled regime, representing the intrinsic losses of the unloaded resonator. The structure does not have 100% contrast because of the not perfect mode matching between the resonator mode and the input light. The contrast can be improved by further prism shape optimization. The nonzero background results from the light that is not coupled to the resonator and is reflected from the prism surface.

We followed this simple model and were able to achieve controllable coupling by changing the air gap between the prism and the SNAP cavity. The coupling efficiency was better than 40% (Fig. 7b). It corresponds to ∣*ξ*_2_∣^2^ ≃ 0.4, if we link the observation with the theoretical description presented above. The coupling can be further improved by optimizing the prism curvature, which requires accurate determination of the SNAP mode geometry. This is the important observation of the unloaded and nearly critically coupled SNAP cavity using prism couplers.

**Fig. 8:**
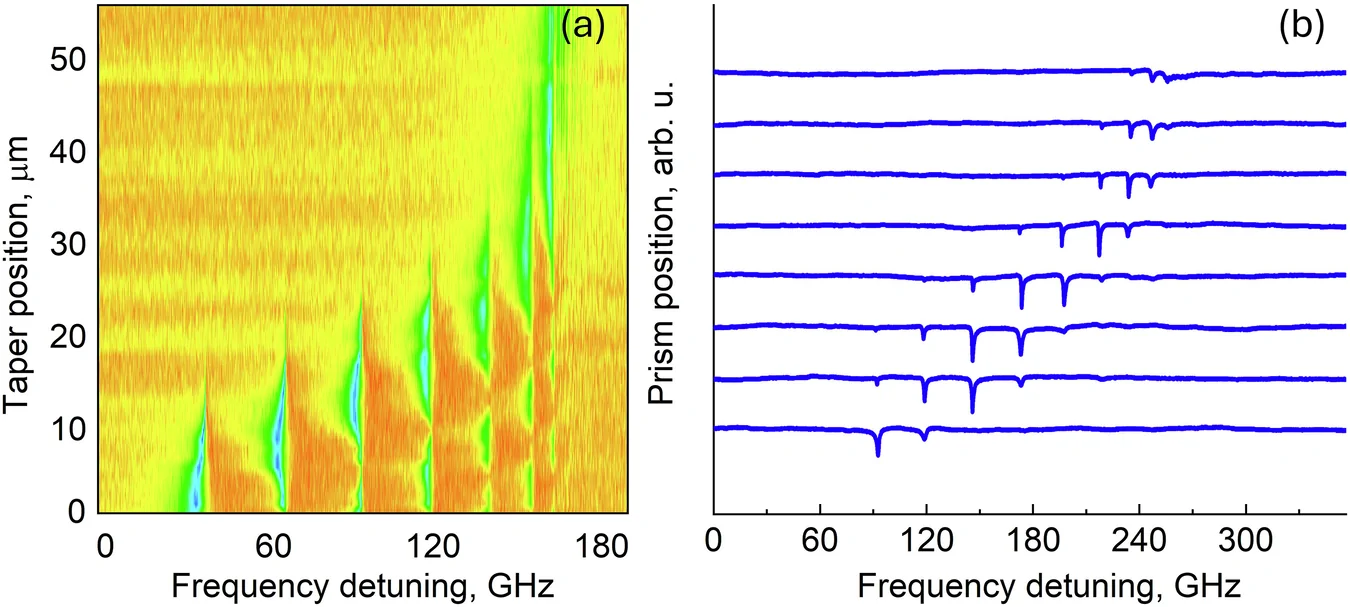
Optical spectra. The spectra were measured at different points of the resonator **a** using fiber taper, and **b** prism coupler. The contrast of the spectra is not normalized and is used exclusively for illustration of the frequencies of the modes.

Using the prism coupler, we were able to map the SNAP spectrum and compare it with the spectrum taken with a tapered fiber coupler (Fig. 8). To take the spectrum, we attached the prism to an automated translation stage and moved the prism along the optical waveguide while scanning the laser. Several scans are shown in Fig. 8. The first scan starts at the center of the SNAP structure. The spectrum of the same structure was also taken using a tapered waveguide (Fig. 8a), and the results matched. The observations validated the prism coupling technique for SNAP. We also can see that the prism coupling can have a better measurement resolution since, unlike the fiber taper that touches the SNAP surface, the prism allows keeping a finite airgap, reducing the coupling efficiency, leading to observation of the spectrally narrower optical modes. While fiber tapers generally allow achieving nearly any desired coupling efficiency through proper positioning of the waveguide, the prism coupler enables this to be done in a more controllable manner within the compact packaged configuration studied here.

### On SNAP integration feasibility

**Fig. 9:**
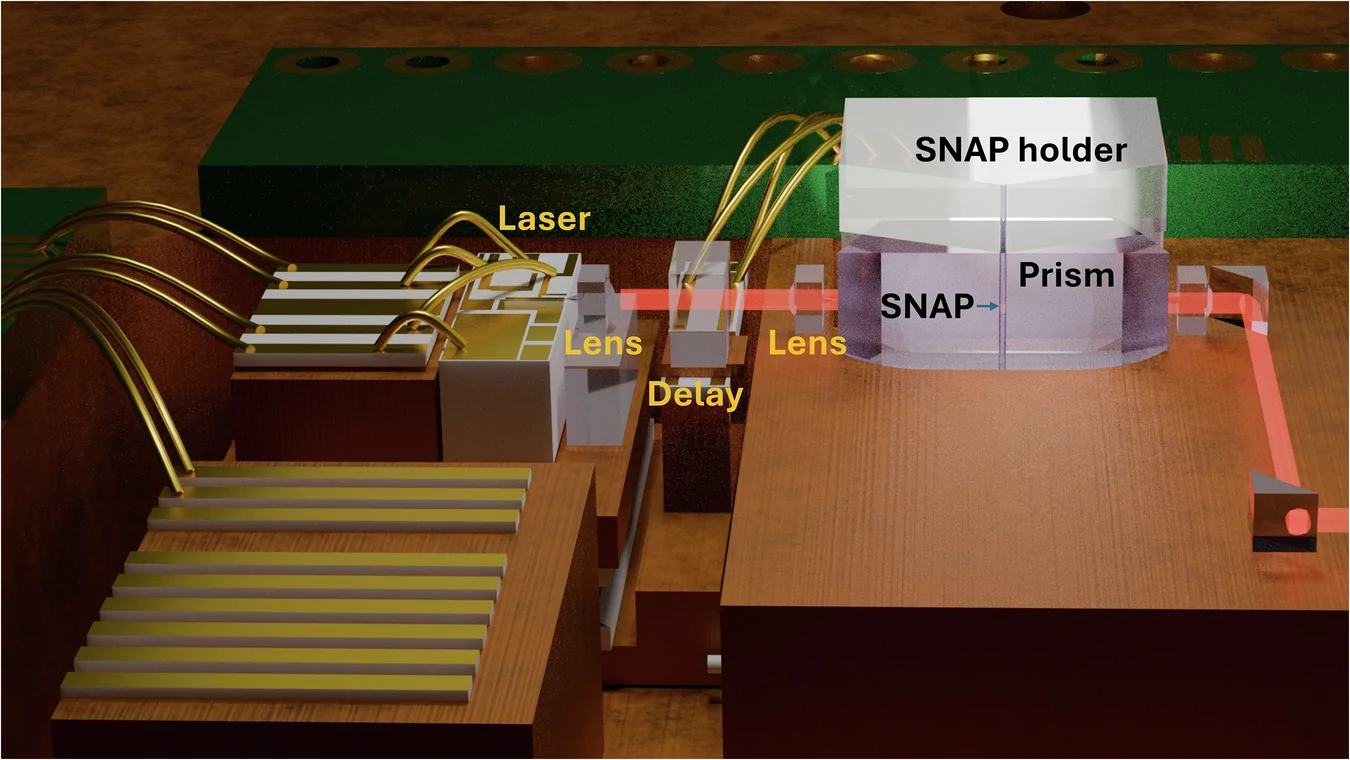
Design of a packaged SNAP structure. A design of a laser chip and micro-optical components co-packaged with a SNAP holding structure integrated with a prism coupler.

The main advantage of the SNAP structure integrated with a prism coupler is its suitability for a tight packaging. The prism is a solid structure and if its size is small enough it has high mechanical stability. The same is related to the optical fiber supporting SNAP. We validated this statement numerically by designing a fused silica holder of a SNAP resonator integrated with a semiconductor laser on submount (Fig. 9).

The SNAP fused silica holder has 5 mm height, 5 mm width, and 6 mm depth. It keeps the SNAP structure 1 μm from the prism surface. The 5 mm long optical fiber supporting SNAP is attached to two protrusions on top and the bottom of the holder and maintains a fixed 1 μm air gap from the prism surface. This is an approximate value of the air gap needed to support loaded Q-factor of 10^6^.

The rest of the optical components shown in Fig. 1 are utilized to couple light from the laser to the SNAP resonator. They include two microlenses and a phase shifter needed for potential self-injection locking of the laser chip to the SNAP structure.

**Fig. 10:**
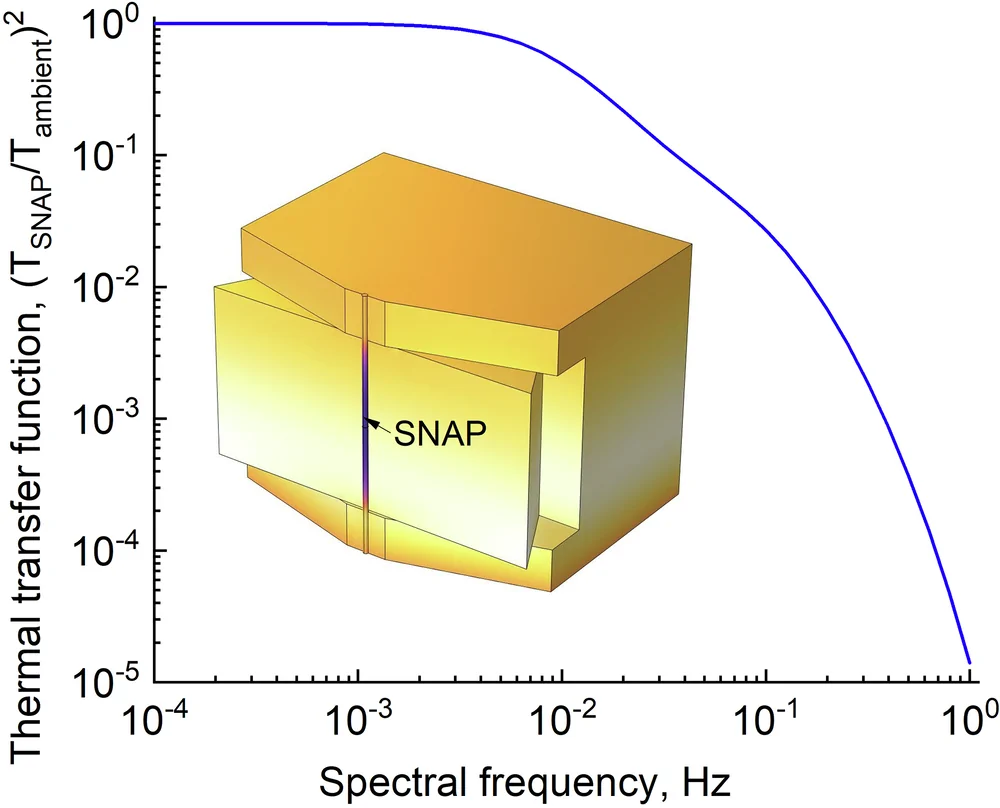
Thermal transfer function of the SNAP-prism assembly. Numerical simulation of the thermal transfer function of the SNAP structure mounted on a platform with changing temperature. The thermal isolation equivalent time approaches 100 s. Inset illustrates thermal isolation of SNAP at 100 mHz.

To verify the thermal and mechanical stability of the design we performed numerical simulations using a finite-element commercial solver. Firstly, we found the thermal transfer function of the SNAP build (Fig. 10). We assumed that the SNAP structure is mounted on a platform with changing temperature and identified how much the SNAP resonator is isolated from the external thermal variations. At lower spectral frequencies, the temperature of the platform is transferred to the temperature of the resonator without changes. Between approximately 5 mHz and 100 mHz the SNAP suspension starts isolating the resonator from the ambient temperature fluctuations. The isolation increases dramatically at spectral frequencies above 100 mHz due to salient SNAP geometry represented by the low heat-conducting fused silica fiber attached in two points to the holders.

**Fig. 11:**
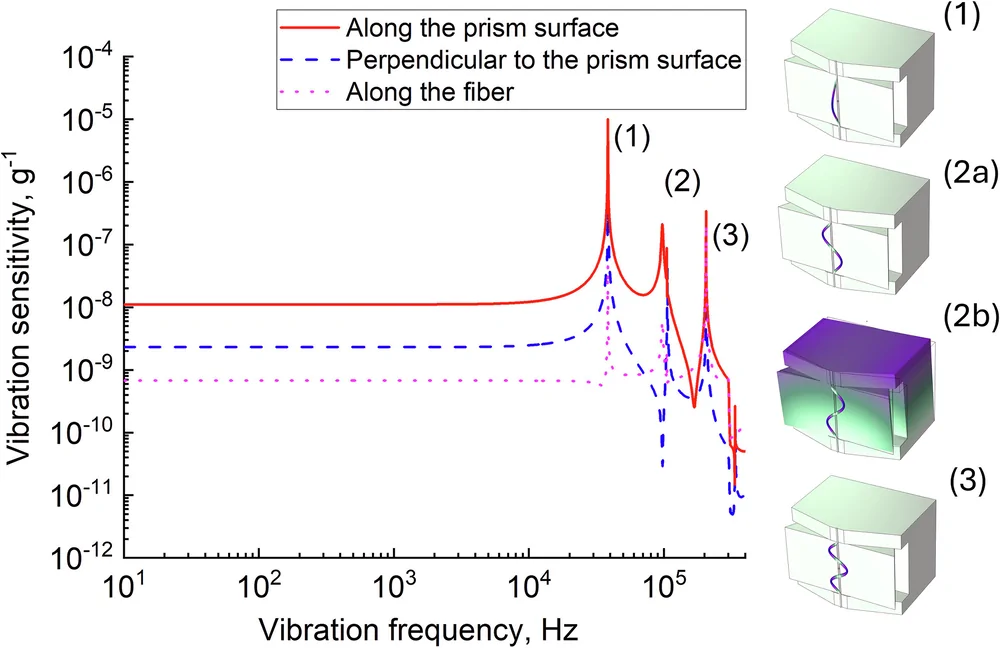
Vibration sensitivity of the SNAP-prism assembly. Numerical simulation of the vibration sensitivity of the SNAP structure mounted on a vibrating platform. The first mechanical mode (1) is identified at 38 kHz. The second vibration mode is a 107 kHz and 113 kHz doublet defined by the SNAP fiber (2a) as well as SNAP mounting structure (2b), respectively. The third mechanical mode is at 207 kHz (3).

We also studied numerically the vibration sensitivity of the SNAP-prism assembly. To do it we assumed that the assembly is attached to a plane surface that experiences random vibrations in a broad spectral range and evaluated the shift of the SNAP relative optical frequency as a function of the frequency of the vibration. The result of the simulation is illustrated by Fig. 11. The structure has high-frequency and high-quality-factor mechanical modes that are usually harmless for devices deployed on moving platforms. At low frequencies, the vibration sensitivity of the structure does not exceed 10^−8^. This is an acceptable value for the majority of micro-optical systems.

It is also important to know how much the vibration changes the air gap between the prism and the SNAP created to support optimally loaded resonances. We evaluated the maximum DC shift of the gap to be 2.6 × 10^−10^ m/g. Assuming that the gap is 10^−6^ m, we see that the relative gap change is 2.6 × 10^−4^/g. The maximum relative mode frequency shift of the SNAP resonance due to gap change from 10^−6^ m to zero is less than the half-width at the half maximum of the mode at zero air gap. In the case of SNAP it is less than 1 GHz, in accordance with our direct measurements. It means that the relative frequency shift due to loading change does not exceed 10^−8^/g. This is approximately equal to the vibration sensitivity due to the deformation of the resonator.

Therefore, in accordance with our numerical simulations, the SNAP structure can be packaged in a compact form factor with a micro-prism evanescent field coupler that can successfully withstand standard fluctuations of the ambient temperature as well as reasonable mechanical vibrations.

## Conclusion

In this work, we report on direct visualization of the SNAP cavities and the development of efficient coupling of light to SNAP cavities with an optical prism. We experimentally visualize surface axial photonic structures whose total radial height is approximately 0.2 nm. The underlying axial potential landscape of a SNAP resonator has been resolved directly rather than inferred from spectral data. This capability provides unprecedented experimental access to a class of nanoscale features that governs the behavior of SNAP devices. The work also presents the design, optimization, and implementation of a previously unavailable type of prism coupler. Its geometry enhances sensitivity to nanoscale surface perturbations on curved, non-planar microresonator surfaces, capabilities that standard planar or fiber-taper couplers do not provide. This is an enabling technology for stabilizing and packaging SNAP devices since longstanding stability limitations of tapered-fiber coupling have been a barrier to deploying SNAP resonators in real compact devices. By demonstrating robust, reproducible coupling with the optimal prism design, we remove a key obstacle that has confined SNAP to laboratory studies for more than a decade. The prism coupling is promising for future integration of the cavities in the mechanically rigid configurations.

## Data Availability

Data underlying the results presented in this paper are not publicly available at this time but may be obtained from the authors upon reasonable request.
